# Synthesis and characterization of Schiff's bases of sulfamethoxazole

**DOI:** 10.1186/2191-2858-4-1

**Published:** 2014-02-28

**Authors:** Zainab Hussain, Emad Yousif, Ahmed Ahmed, Ali Altaie

**Affiliations:** 1 Department of Chemistry, College of Science for Women, Baghdad University, Baghdad, Iraq; 2 Department of Chemistry, College of Science, Al-Nahrain University, Baghdad, Iraq

**Keywords:** Schiff bases, Sulfamethoxazole, Synthesis

## Abstract

**Background:**

Schiff's bases are excellent ligands which are synthesized from the condensation of primary amines with carbonyl groups.

**Findings:**

The classical reaction for the synthesis of Schiff's bases in an ethanolic solution and glacial acetic acid as a catalyst was followed in the synthesis of substituted sulfamethoxazole compounds.

**Conclusions:**

Some Schiff's bases containing sulfamethoxazole nucleus have been synthesized and characterized. The present compounds are hoped to be applied in the photostability of PVC.

## Findings

### Background

Schiff's bases are an important class of organic compounds [1]. They were first reported by Hugo Schiff in 1864 [2]. Schiff's bases are condensation products of primary amines with carbonyl compounds. The common structural feature of these compounds is the azomethine group with the general formula RHC = N-R_1_, where R and R_1_ are alkyl, aryl, cycloalkyl, or heterocyclic groups [1]. Structurally, a Schiff's base (also known as imine or azomethine) is a nitrogen analogue of an aldehyde or ketone in which the carbonyl group (>C = O) is replaced by an imine or azomethine group. Schiff's bases have also been shown to exhibit a broad range of biological activities, including antifungal, antibacterial, antimalarial, antiproliferative, anti-inflammatory, antiviral, and antipyretic properties [3,4]. Imine or azomethine groups are present in various natural, naturally derived, and nonnatural compounds. The imine group present in such compounds has been shown to be critical to their biological activities [5-7]. Schiff's bases are important compounds owing to their wide range of industrial applications [8]. Schiff's bases are used in the photostabilization of poly(vinyl chloride) polymers against photodegradation by ultraviolet radiation [9-11] and are also used to improve poly(methyl methacrylate) from degradation [12] and to prevent polystyrene from photodegradation by their addition to polymer films [13,14].

### Methods

Fourier transform infrared (FTIR) spectra were registered on a SHIMADZU (8300, Kyoto, Japan) infrared spectrophotometer, using KBr discs. Proton nuclear magnetic resonance (^1^H-NMR; 600 MHz) spectra were obtained at room temperature with Bruker equipment (Madison, WI, USA) using TMS as an internal standard in dimethyl sulfoxide (DMSO). Melting points were recorded using hot-stage Gallenkamp melting point apparatus (Loughborough, UK) and were uncorrected. Analytical grade chemicals (BDH, G.C.C., Hopkin & William Corporation, Poole, UK) were used throughout the project.

### Results and discussion

**Scheme 1:**
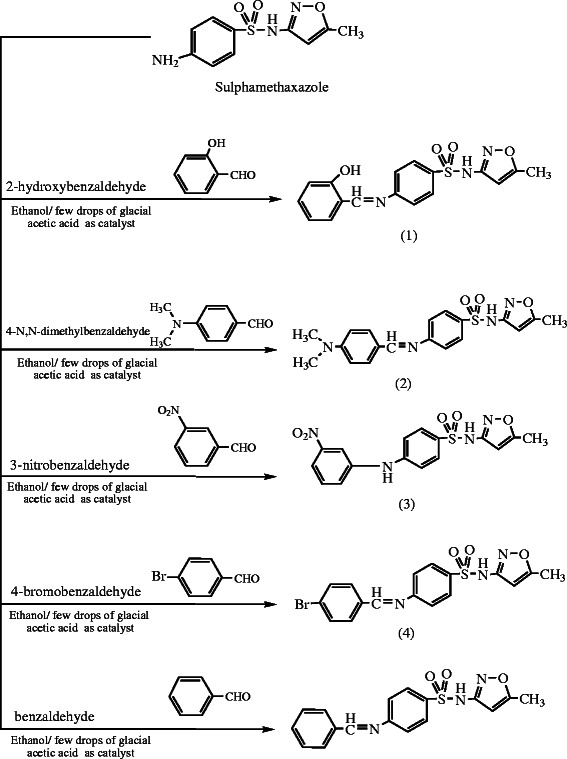
**Scheme of the preparation of Schiff's bases from sulfamethoxazole.** (1) 4-[(2-Hydroxy-benzylidene)-amino]-*N*-(5-methyl-isoxazol-3-yl)-benzene sulfonamide. (2) *N*-(5-Methyl-isoxazol-3-yl)-4-[(4-nitroox-benzylidene)-amino]-benzene sulfonamide. (3) 4-[(4-Dimethylamino-benzylidene)-amino]-*N*-(5-methyl-isoxazol-3-yl)-benzene sulfonamide. (4) 4-[(4-Bromo-benzylidene)-amino]-*N*-(5-methyl-isoxazol-3-yl)-benzene sulfonamide. (5) (*N*-(5-Methyl-isoxazol-3-yl)-4-[(3-nitro-benzylidene)-amino]-benzene sulfonamide.

The synthesis of Schiff's bases with different specific aldehydes in ethanol as a solvent and catalyst (glacial acetic acid) resulted in five new series of Schiff's bases with the general formula RHC = N-R_1_. Here R_1_ = sulfamethoxazole and R = benzaldehyde, 4-bromobenzaldehyde, 2-hydroxybenzaldehyde (salicylaldehyde), 4*-N*,*N-*dimethylbenzaldehyde, and 3-nitrobenzaldehyde were synthesized by the reaction of sulfamethoxazole and substituted aldehydes in ethanol (Scheme 1). Such compounds were characterized by different physicochemical techniques like melting point, elemental analysis, FTIR spectroscopy, and multinuclear NMR (^1^H).

### Physical properties of the prepared Schiff's bases

The physical properties of sulfamethoxazole and its derivatives including melting point, color, and elemental analysis are tabulated in Table 1.

**Table 1:** Physical data of the prepared compounds

**Compounds**	**Color**	**MP (°C)**	**Elemental analysis, theoretical (actual)**				
			**% C**	**% H**	**% N**	**% O**	**% S**
(1)	Yellow	190 to 192	57.13	4.23	11.76	17.91	8.97
			(57.23)	(4.88)	(10.39)	(16.88)	(9.11)
(2)	Brown	148 to 150	60.85	6.23	13.52	11.58	7.74
			(59.82)	(6.43)	(13.63)	(12.09)	(8.10)
(3)	Orange	122 to 124	52.84	3.65	14.50	20.70	8.30
			(53.88)	(3.22)	(13.91)	(19.33)	(8.21)
(4)	Light yellow	142 to 143	48.58	3.36	10.00	11.42	7.63
			(49.59)	(3.51)	(9.33)	(10.21)	(8.33)
(5)	Orange	110 to 112	59.81	4.43	12.31	14.06	9.39
			(69.88)	(3.89)	(12.49)	(14.63)	(10.21)

The structure of the prepared Schiff's bases was confirmed by infrared spectroscopy. The FTIR spectra of sulfamethoxazole and its prepared compounds, as shown in Table 2, showed that the band of NH_2_ was found in sulfamethoxazole in the location 3,298 cm^−1^ and then vanished. After that, the band of NH appeared in the prepared Schiff's bases with different shifting from 3,250 to 3,287 cm^−1^. The band of C = N for imine stretching vibration was also not found in sulfamethoxazole, and it appeared in the prepared Schiff's bases with shifting from 1,603 to 1,650 cm^−1^. Also, the band of C = N for ring stretching vibration shifted from 1,620 cm^−1^ in sulfamethoxazole to 1,615 to 1,630 cm^−1^ in the prepared compounds.

**Table 2:** FTIR spectroscopy for sulfamethoxazole and its derivatives

**Compounds**	***υ *** **(NH**_**2**_**) (cm**^**−1**^**)**	***υ *** **(N-H) (cm**^**−1**^**)**	***υ *** **(C = N) imine (cm**^**−1**^**)**	***υ *** **(C = N) ring (cm**^**−1**^**)**
Sulfamethoxazole	3,298	-	-	1,620
(1)	-	3,250	1,650	1,616
(2)	-	3,266	1,603	1,620
(3)	-	3,290	1,650	1,600
(4)	-	3,270	1,650	1,630
(5)	-	3,287	1,633	1,615

The ultraviolet-visible spectrophotometry technique is used to characterize sulfamethoxazole and its derivatives in DMSO as a solvent. The ultraviolet-visible electronic spectra of the prepared Schiff's bases showed absorption bands that could be attributed to π → π* electronic transitions; these transitions are assigned in relevance to the structures of the compounds. The electronic spectrum of sulfamethoxazole shows a band at the wavelength 280 nm; this may be attributed to the π → π* electronic transition. In the prepared Schiff's bases, the bands shifted to wavelengths different from that of the corresponding band in sulfamethoxazole, as shown in Table 3, which appear in the wavelength range between 270 and 360 nm. These transitions may be attributed to π → π* and *n* → π* electronic transitions.

**Table 3:** UV spectroscopy for sulfamethoxazole and its derivatives

**Compounds**	**Absorption bands (nm)**	**Assigned transition**
Sulfamethoxazole	280	π → π*
(1)	282, 310	π → π*, *n* → π*
(2)	250, 360	π → π*, *n* → π*
(3)	270	π → π*
(4)	271	π → π*
(5)	270	π → π*

The ^1^H-NMR spectrum of compound (1) showed the following characteristic chemical shifts (DMSO as a solvent): the singlet signal at *δ* = 2.212 ppm suggested the attribution of the protons of the CH_3_ group, the singlet signal at *δ* = 6.029 ppm suggested the attribution of the proton of CH of the isoxazole ring, the multiplet signal at *δ* = 6.743 to 7.768 ppm suggested the attribution of the protons of two aromatic benzene rings, the singlet signal at *δ* = 8.764 ppm suggested the attribution of the proton of the CH = N group, the singlet signal at *δ* = 9.352 ppm suggested the attribution of the proton of the NH group, and the singlet signal at *δ* = 10.525 ppm suggested the attribution of the proton of the OH group.

Also, the ^1^H-NMR spectrum of compound (5) showed the following characteristic chemical shifts (DMSO as a solvent): the singlet signal at *δ* = 2.200 ppm suggested the attribution of the protons of the CH_3_ group, the singlet signal at *δ* = 5.833 ppm suggested the attribution of the proton of CH of the isoxazole ring, the multiplet signal at *δ* = 6.789 to 7.749 ppm suggested the attribution of the protons of two aromatic benzene rings, the singlet signal at *δ* = 8.698 ppm suggested the attribution of the proton of the CH = N group, and the singlet signal at *δ* = 9.602 ppm suggested the attribution of the proton of the NH group [15], as shown in Table 4.

**Table 4:** ^**1**^**H-NMR data of sulfamethoxazole and its derivatives**

**Compounds**	**CH**_**3**_	**C-H isoxazole ring**	**Aromatic benzene rings**	**Imine**	**N-H**	**OH**
(1)	2.212	6.029	6.743 to 7.768	8.764	9.352	10.525
(5)	2.200	5.833	6.789 to 7.749	8.698	9.602	-

### Experimental

A solution of sulfamethoxazole (0.001 mol) in absolute ethanol (30 ml) was slowly added to a solution of aldehyde (0.001 mol) in absolute ethanol (20 ml). The stirred reaction mixture was refluxed for 12 h. After cooling, a precipitate was formed which was collected by filtration, then washed with cold ethanol, and recrystallized from ethanol.

### Conclusion

Five Schiff's bases: (1), (2), (3), (4), and (5), were synthesized as derivatives of sulfamethoxazole and characterized by UV, FTIR, and ^1^H-NMR spectroscopies and elemental analysis (CHNS).

## Competing interests

The authors declare that they have no competing interests.
